# Supplementary figures and images for: XRN2 suppresses aberrant entry of tRNA trailers into argonaute in humans and Arabidopsis
Source: PLoS Genet. 2023 May 5;19(5):e1010755. doi: 10.1371/journal.pgen.1010755 (PMC10191329; doi:10.1371/journal.pgen.1010755)

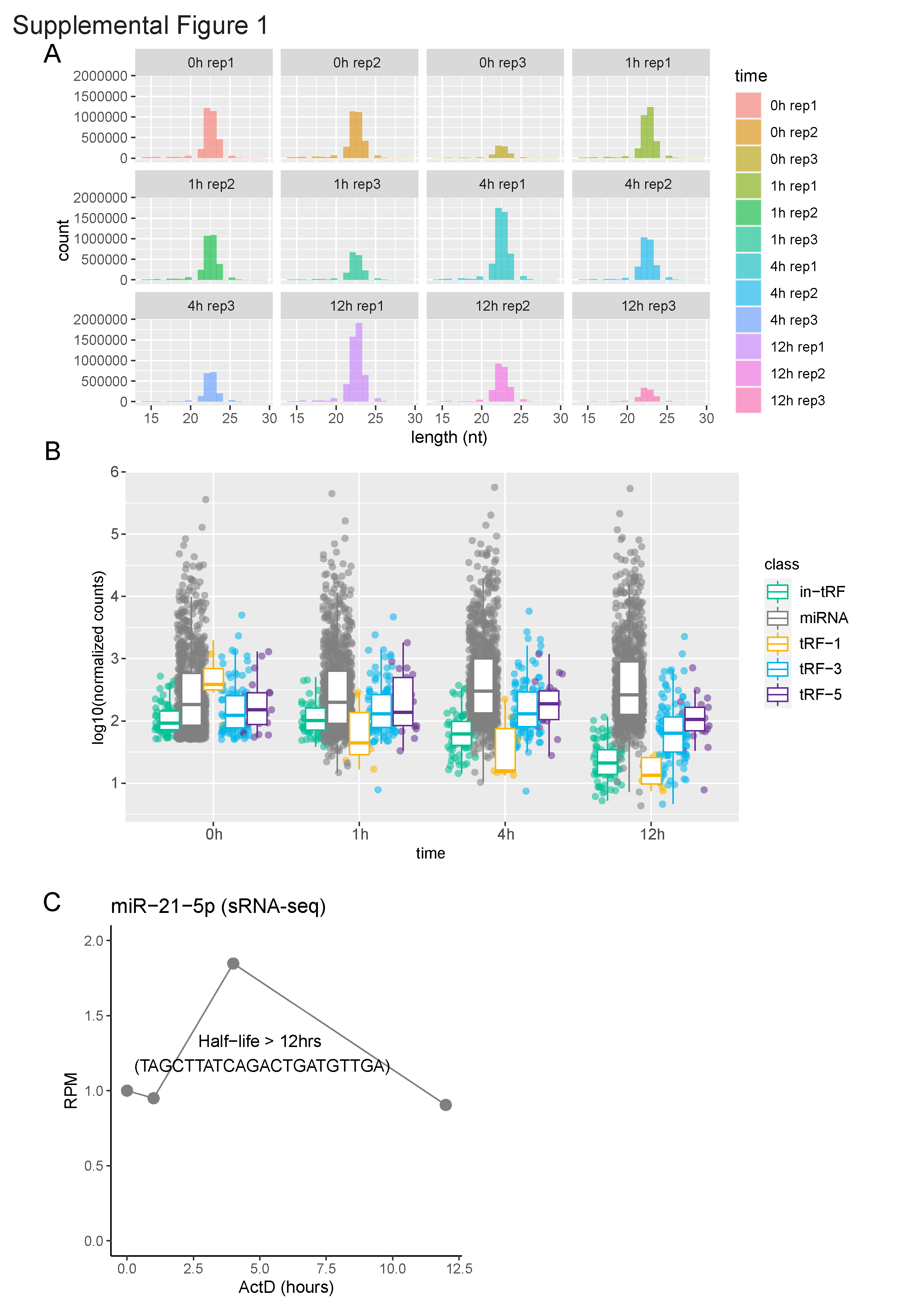

Supplement: S1 Fig — B) tRF-1s are an abundant class of sRNAs at steady state. Shown are the normalized count distributions for each small RNA class at each actD time point in HDMYZ cells. tRF-1s are most abundant at time of generation (median abundance: 388.61 RPM). C) Decay curve for miR-21-5p in HDMYZ cells (sRNA-seq). (TIF) [file pgen.1010755.s001.tif]

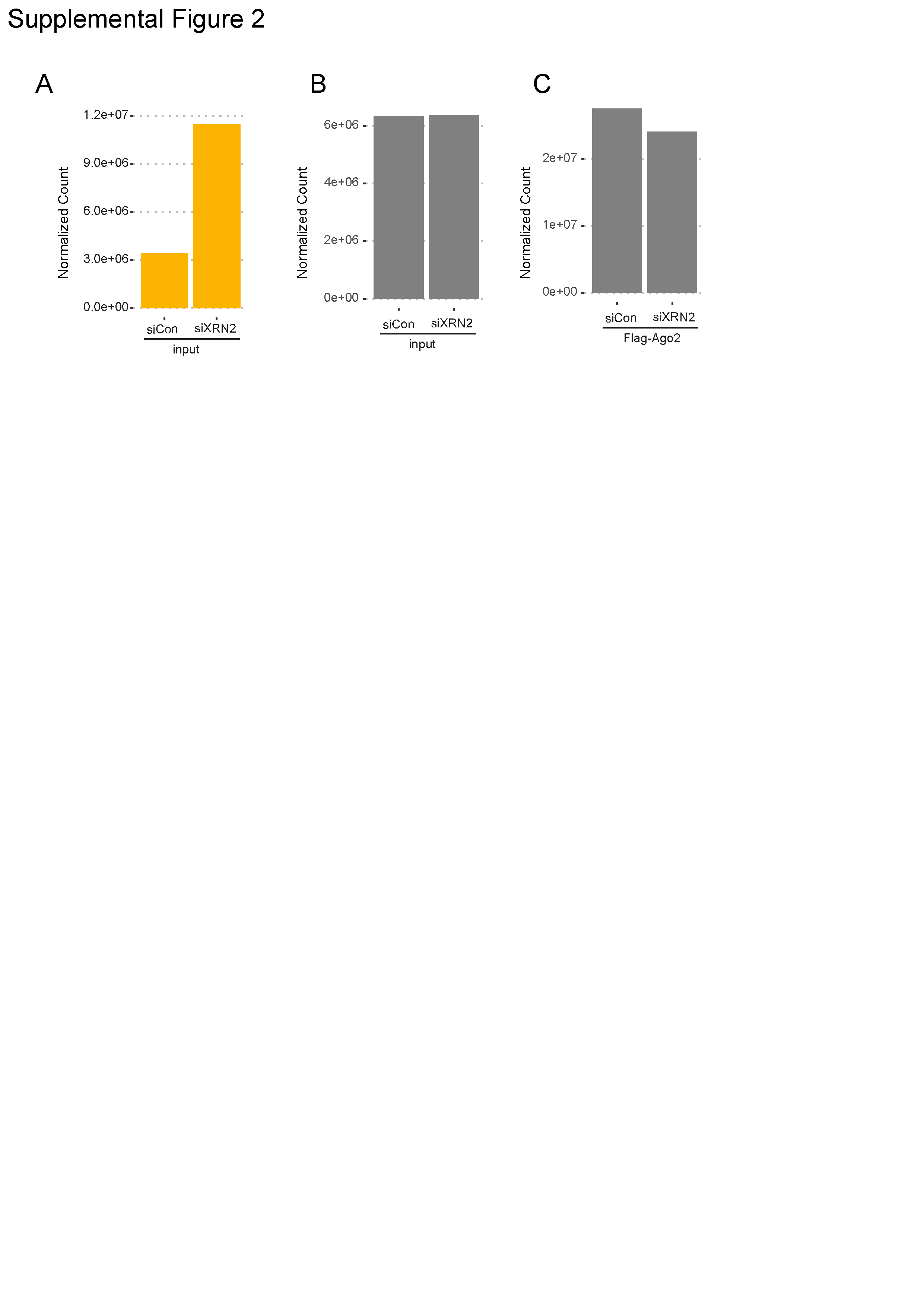

Supplement: S2 Fig — C) Bar plot of total number of miRNAs associated with Ago2 after siCon or siXRN2 (TIF) [file pgen.1010755.s002.tif]

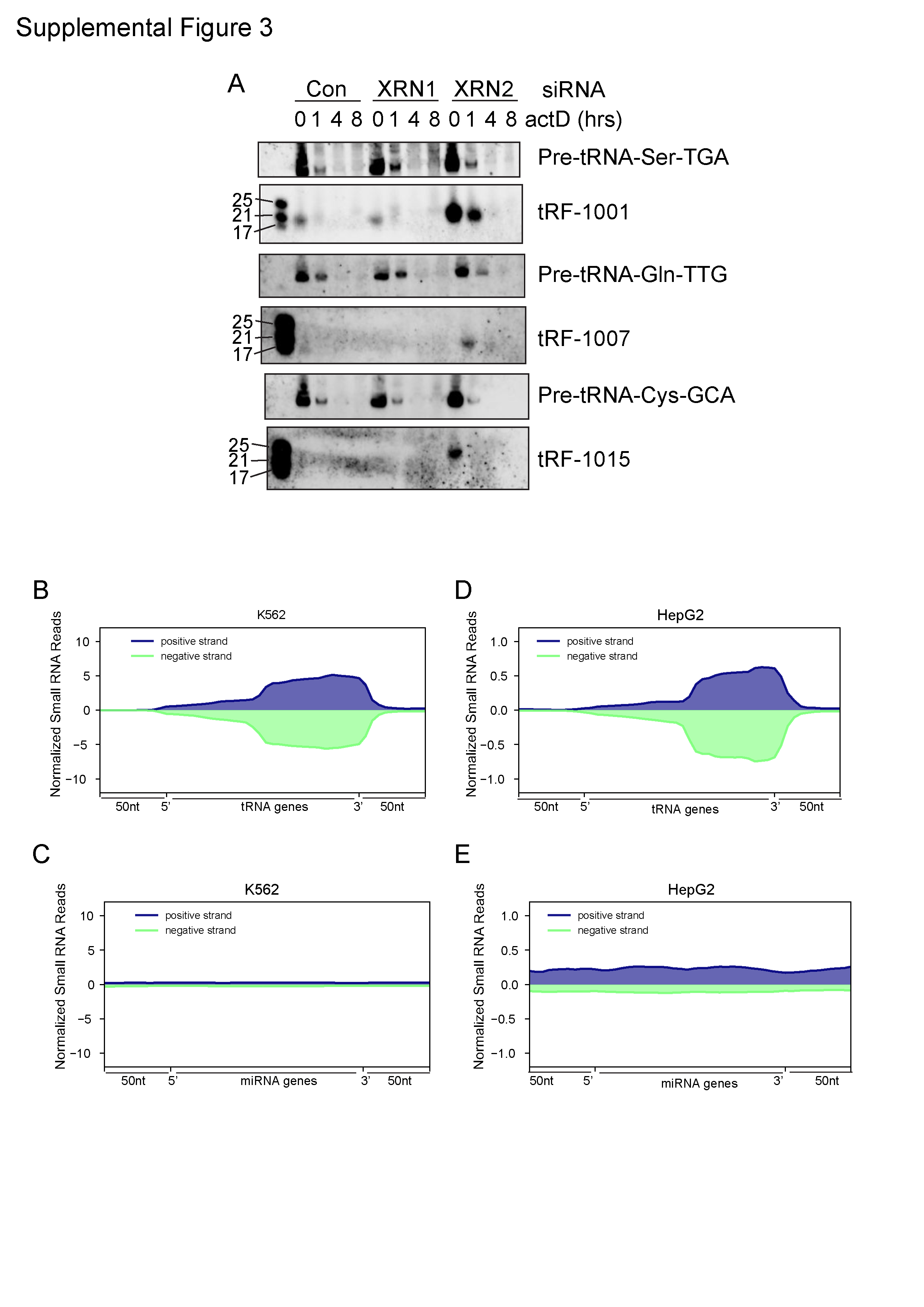

Supplement: S3 Fig — Northern probes are complementary to the tRF-1 sequence. B,C) ENCODE XRN2 eCLIP metagene plot for tRNA and miRNA genes, respectively in K562 cells. D,E) Same as in B & C, except in HepG2 cells. (TIF) [file pgen.1010755.s003.tif]
