# Supplementary material for: XRN2 suppresses aberrant entry of tRNA trailers into argonaute in humans and Arabidopsis
Source: PLoS Genet. 2023 May 5;19(5):e1010755. doi: 10.1371/journal.pgen.1010755 (PMC10191329; doi:10.1371/journal.pgen.1010755)
Supplement: Attachment — Submitted filename: 2022-12-22_PGENETICS-D-22-01273_reviewer.pdf [file pgen.1010755.s007.pdf]

The tRNA-derived sRNAs or tRNA fragments (tRFs) have similar sizes to that of miRNAs, but not all the tRFs could be loaded into AGO proteins. Little has been known about how AGO proteins choose specific tRFs over others. In this manuscript, Wilson et al. found tRF-1s are degraded by exoribonuclease XRN2, which prevents their loading into AGO both in human and Arabidopsis.

Overall speaking, the study is interesting and elaborates the relationship between stability of tRFs and their enrichment in RISC. The main approach includes sequencing analysis of small RNA libraries prepared from different treatments (i.e., upon XRN2 depletion). The study fits into the scope of "PLOS Genetics"; and has general interest in abroad audience. The results are very important for RNA field, especially for small RNA research. However, there are rooms for improvement. First, the study appears to lack mechanistic insight into how XRN2 selects tRF1 for degradation; Second, what is the physiological significance for such a regulation? In other words, in what kinds of natural conditions, tRF1 can be loaded into RISC?

A few other points for revision or discussion:

1. It would be much convenient for the readers if the authors could draw a carton/schematic to show tRNA processing in Figure 1A.
2. For Figure 1G, tRF-1001 expression level is relative to miR-21-5p. Why miR-21-5p is chose to perform normalization? How about the relative expressions in Figure 1D/E/F? Are they also relative to miR-21-5p, and why?
3. In Figure 3A, 1h actD treatment will delete all pre-tRNA serTGA in siCon. But in Figure 3C, after 1h actD treatment, pre-tRNA serTGA can still be detected. How to explain this contradiction?
4. Figure 3D is only one example of XRN2 binding and not really that convincing. Please check XRN2 binding profile around all pre-tRNAs using the eCLIP data.
5. In Figure 3E, expressing catalytic dead XRN2 accumulate more tRF-1s than shXRN2, how to explain this?
6. "1 was a miRNA, 1 mapped to a piRNA locus, 1 was a misc-tRF, 3 were intron-tRFs, and 25 were tRF-1s." It would be better to use word rather than number, if the single digit is subject.
7. Since you introduce Figure 4E before Figure 4D, it would be better to change the order of 4E and 4D.
8. For Figure 5 legend, it should be "E, G) Scatter plot" rather than "E, F) Scatter plot".
9. Figure 6 model was not discussed in the body paragraphs.
