# Supplementary material for: XRN2 suppresses aberrant entry of tRNA trailers into argonaute in humans and Arabidopsis
Source: PLoS Genet. 2023 May 5;19(5):e1010755. doi: 10.1371/journal.pgen.1010755 (PMC10191329; doi:10.1371/journal.pgen.1010755)
Supplement: Attachment — Submitted filename: XRN2_tRF1_reviewerComments_BW_02252023.docx [file pgen.1010755.s008.docx]

We appreciate the reviewers enthusiasm for our manuscript. We agree that several of their suggestions will make the manuscript stronger and more clear to readers. Please see our responses below.

**Reviewer #1: In this manuscript, the authors analyzed the mechanism degrading tRNA trailers (tRF-1s), which are byproducts of tRNA processing and have size similar to that of miRNAs. The authors found that the tRF-1s have short half-life and depletion of the 5’ to 3’ exoribonuclease XRN2 and XRN1 increases the stability of tRF-1s in human cell lines. In addition, knockdown of XRN2 is sufficient to increase the half-life of tRF-1s. Induced expression of wild-type XRN2, but not inactive XRN2, decreases the stability of tRF-1s in the knockdown line of XRN2. Moreover, XRN2 binds some tRF-1s. These data show that XRN2 degrades tRF-1s. The authors further showed that the amount of tRF-1s are increased in AGO IPs in the knockdown lines of XRN2 relative to wild-type. Similarly in Arabidopsis, defection in XRNs also increases the abundance of tRF-1s in total RNAs, and in AGO1/2-IPs. Based on these observations, the authors proposed that XRN2 degrades tRF-1s to exclude them from AGOs. The finding that XRN2 degrades tRF-1s is novel.**

**Some suggestions are:**

**It seems that tRF-1001 is more abundant other tRF-1s (instruction) in wild-type. why this one cannot be loaded into AGOs in wild-type, although knockdown XRN2 leads to its accumulation in AGO?**

We thank the review for highlighting this key point. We were also perplexed as to why stabilized tRF-1001 cannot enter AGO efficiently. We suggest two different reasons for this observation:

1-tRF-1001 is a relatively short small RNA with length ranging from 17 to 19 nucleotides depending on the length of the poly-U tail. This length may not be optimal for AGO loading, as miRNAs are the most efficiently loaded small RNAs and have an average length of about 22 nucleotides.

2-tRF-1001 has been shown to interact with SSB/La so this interaction might prevent AGO loading (Cho et al 2019).

We have added these points to the discussion section:

“…tRF-1001 is bound by SSB/La, which leads to SSB/La sequestration from Hepatitis C virus (HCV) and blunts HCV protein synthesis [[69]](https://paperpile.com/c/xpEjkr/Jd8N). SSB/La binding to tRF-1001 may be another mechanism that specifically precludes tRF-1001 entry into Ago2. Another reason some tRFs may be loaded into Ago2 more efficiently than others relates to tRF length. tRF-1001 is about 17-19 nucleotides in length, which may be a less than optimal length for Ago2 loading, while tRF-1015 is 23 nucleotides and loads much more efficiently.”

**Figure 1A, why counts are less at 4 and 8 hours than at 12 hours? Please provided detailed description in legends.**

We apologize for the confusion regarding Figure 1A (now Figure 1B). Figure 1B is showing the distribution of half-lives across all small RNAs in HDMYZ cells. There are far fewer small RNAs that have halflives of 4 and 8 hours than of 12 hours. We have changed the y axis to “number of short RNAs with indicated halflife”. The high number at 12hrs is largely driven by miRNAs, which have halflives longer than 12 hours. We have clarified this in the manuscript.

**Fig1b, tRF-leader also has short half-life, Does XRN2 affect its half-life? The tRF-leader is missing in fig 2C.**

There was only one tRF-leader that was captured in this small RNA sequencing library. The size ranges of tRF-leaders are less than standard small RNA sequencing size selection cutoffs (~15nts) and so are not accurately captured by sRNA seq. Thus we cannot comment on tRF-leader regulation by XRN2. We have clarified this in the manuscript:

“There was only one tRF-leader that was captured in this small RNA sequencing library. The size ranges of tRF-leaders are less than standard small RNA sequencing size selection cutoffs (~15nts) and so are not accurately captured by sRNA seq. Therefore, in this paper we have mainly focused on other highly abundant tRFs.”

**Fig 3c, is this result statistically significant?**

**polIII should be Pol III? Please go through the manuscript**

We have now changed “polIII” to “Pol III”

The result is reproducible in two independent experiments, and statisticians recommend that we do not calculate a p value for n=2. However, for the reviewers satisfaction, we have performed a two sided t-test for the 1hr time point: p-value = 0.05114

**Fig 4E legends, should be “altered changes in siXRN2 relative to siCons?”.**

We thank the reviewer for pointing this out. We have changed this in the manuscript.

**Fig 6, the model proposed XRN2 affects both tRF-1s and tRF-introns. Do the authors have data support from Arabidopsis? A brief description for the model should be provided.**

There are 22 unique tRNA introns in Arabidopsis. The average tRNA intron length is about 13, which is lower than the minimum cutoff that was captured in that small RNA library. Manual inspection of the few introns that were greater than or equal to 14nts identified a total of 3 reads for the two wildtype small RNA sequencing libraries, perhaps due to 5’-hydroxyls and 3’ cyclic phosphates preventing cloning of more tRF-introns during library preparation. Future studies will have to include specific considerations at the library preparation step to ensure adequate capture of tRNA introns in Arabidopsis. However, even if the tRNA introns are adequately cloned, their short length may make subsequent mapping to the genome difficult. We have added this discussion to the manuscript. We have also added a description of the model outlined in Figure 6 to the figure legends:

“…we focused on the relationship between tRF-1s, -5s, -3s, -leaders, and misc-tRFs and XRN inhibition in *A. thaliana*. Very few intron-tRFs were detected in the wildtype small RNA libraries. Manual inspection of the few introns that were greater than or equal to 14nts identified a total of 3 reads for the two wildtype small RNA sequencing libraries, perhaps due to 5’-hydroxyls and 3’ cyclic phosphates preventing cloning of more tRF-introns during library preparation.”

“**Figure 6. Proposed model for XRN2 mediated prevention of tRF-1 entry into the miRNA effector pathway.** In mammalian cells, tRF-1s are rapidly degraded by XRN2 which prevents tRF-1 entry into the miRNA effector pathway. intron-tRFs are also significantly increased when XRN2 is deleted in mammalian cells, suggesting that XRN2 may also prevent their accumulation in Ago2. We find evidence of a similar mechanism in *A. thaliana*, where XRN inhibition by PAP accumulation results in an increase in tRF-1s associated with Ago1 and Ago2.”

**Reviewer #2: The authors hope to demonstrate that XRN2 selectively degrade tRF-1s thus blocks tRF-1 accumulation in RISC in human and Arabidopsis. While the manuscript provides some interesting observations, I find some the data rudimentary and not strong enough to support the conclusion. I provide some comments below for authors’ improvement**

**In Fig.1B, what does each dot mean? Does each dot represent a specific small RNAs? There should be much more sequences than the dots show. some tRF-1s might be resilient than others? What is the tRNA origin of them? are there any pattern? Also, is there no replication for all the sequencing data? if this is true this is unacceptable.**

We appreciate the reviewers for pointing this out. Each point represents a single sequence. The half-life was calculated based on the average expression of the sequence. There are three biological replicates including 3 sequencing libraries for each time point. We have now made this clear in the methods section:

“HDMYZ cells are an acute myeloid leukemia cell line. Small RNA sequencing data from HDMYZ cells was downloaded from GSE46968. There are three biological replicates for each actinomycin D time point. For actinomycin D small RNA sequencing, if the small RNA decreases to a size below the sequencing library cutoff, these small RNAs will not be cloned and counted. This does not affect the conclusions because the shortening is part of the degradation processes. Arabidopsis small RNA sequencing data was downloaded from GSE133461. There are two biological replicates for each total small RNA sequencing library and three biological replicates for each Ago RNA immunoprecipitation sequencing library.”

There are very few points for tRF-1s in Figure 1B because only a few tRF-1s are detected at the 12 hour time point after actinomycin D treatment. There are many more tRF-1s detected at the one hour time point, and the tRF-1s are ~2-5 fold decreased at this time point. Figure 1C is shown to exemplify this point. We have also added the number of points in each small RNA class for Fig 1B (Now Fig 1C, there are a very large number of miRNAs that have half-life > 12hr).

There was no clear pattern for tRF-1s that remained at 12 hours. The tRF-1s ranged from 14 to 25 nucleotides in length, with an average length of 21 nucleotides. The tRF-1s arose from various parental precursor tRNAs: tRNA-Leu-TAG-3-1, tRNA-His-GTG-1-7, 2 3’-poly-U tail isoforms from tRNA-Val-TAC-1-2, tRNA-Thr-AGT-1-2, tRNA-Asn-GTT-2-2, and tRNA-Ser-GCT-4-1. We have added our speculations regarding the relative stability of these tRF-1s to the discussion:

“Very few tRF-1s are present at the 12 hour actD time point in HDMYZ cells, and there aren’t any clear patterns in regards to tRF-1s with higher stability relative to less stable tRF-1s. Perhaps these tRF-1s are bound by unidentified proteins, interact with other RNAs, or are otherwise somehow protected from fast XRN2 turnover.”

**All sequencing data have two to three replicates and we have added this to the methods section.** We have clarified these points in the manuscript.

**The overall length distribution of different types of small RNAs should be shown, also, what is the effect of actinomycin D on the overall length distribution of all small RNAs? Will there be an overall decreased length of small RNAs? And if a small sequence become shorter, to what extent it will not be counted as a small RNA? These essential data are missing. Suppl Fig.1 showed only one sequencing result? No replication? The sequencing result after actinomycin D treatment should also be shown.**

If the small RNA decreases to a size below the sequencing library cutoff, these small RNA will not be cloned and counted. This does not affect the conclusion because the shortening is part of the degradation processes. We have added this to the text (methods).

For supp fig 1, the average expression is based on sequencing results from 3 biological replicates and sequencing libraries as stated above. We have clarified this in the figure legend.

We have added the average expression and length distribution for all small RNAs after actD treatment for all time points in new supplemental figure 1:

**Supplemental Figure 1.** A) Length distributions of all small RNAs by actD time point. B) tRF-1s are an abundant class of sRNAs at steady state. Shown are the normalized count distributions for each small RNA class at each actD time point in HDMYZ cells. tRF-1s are most abundant at time of generation (median abundance: 388.61 RPM).

**For Fig.2A, Fig.3A-C, the Northern blot data of tRNA and tRF using one probe should not be shown separately, need to be shown on a same exposure to see the relatively intensity. The authors want to hide something? Also, this is a key piece of data showing that XRN1/2, XRN2 depletion increases tRF-1s expression, with this level of quality, it will not support the conclusion. More tRF-1s shown by bioinformatic analysis (e.g., Fig.1b) need to be validated using Northern blot.**

1. Below we have shown three complete NB to show the precursor and tRF on the same blot.
2. We have also published (Su et al 2022, Kuscu et al 2018) similar NB to assure the reviewer that we are not hiding something. In control cells, we usually detect higher signals for tRNA compared to tRF. To prevent over-exposure of tRNA signal, we cut the membrane to probe for tRF and tRNA separately to ensure both can be detected within linear range.
3. We have shown the increase of tRF-1001, tRF-1007, and tRF-1015 after siXRN2 by NB below and in a new supplemental figure 3A. The levels of tRF-1001, tRF-1007, tRF-1015 are increased at the zero hour time point (steady state) by siXRN2.

**Are there any clues what XRN2 selectively degrade tRF-1s? any sequence specificity? Or due to internal/terminal modifications?**

The specificity of XRN2 for tRF-1s likely arises from the fact that tRF-1s are single stranded and have a 5’ phosphate. We have clarified this in the manuscript:

“The specificity of XRN2 for tRF-1s likely arises from the fact that tRF-1s are single stranded and have a 5’ phosphate. Additionally, while our manuscript was under review, David Bentley and colleagues showed that XRN2 associates with tRNA chromatin and degrades tRNA trailers, supporting our findings [[47]](https://paperpile.com/c/xpEjkr/OiGY).”

**Reviewer #3: The tRNA-derived sRNAs or tRNA fragments (tRFs) have similar sizes to that of miRNAs, not all the tRFs could be loaded into AGO proteins. Little has been known about how proteins choose specific tRFs over others. In this manuscript, Wilson et al. found tRF-1s degraded by exoribonuclease XRN2, which prevents their loading into AGO both in Arabidopsis. Overall speaking, the study is interesting and elaborates the relationship between stability of tRFs and their enrichment in RISC. The main approach includes sequencing analysis of small RNA libraries prepared from different treatments (i.e., upon XRN2 depletion). The study fits into the scope of “PLOS Genetics”; and has general interest in abroad audience. The results are very important for RNA field, especially for small RNA research. However, there are rooms for improvement. First, the study appears to lack mechanistic insight into how XRN2 selects tRF1 for degradation; *Second, what is the physiological significance for such a regulation? In other words, in what kinds of natural conditions, tRF1 can be loaded into RISC?***

We thank the reviewer for pointing out our limited discussion on the functional relevance of stabilized tRF-1s and agree that highlighting possible functional implications in our discussion will help guide future studies. We have added in the discussion some possible physiological conditions in which XRN2 activity or levels are altered, and therefore, tRF-1s might become functionally relevant (quoted text below). We highlight heat stress, where XRN2 changes localization within the nucleus. Pathological or pharmacological instances in which XRN2 is very low or inhibited: Cancer vs normal, and conditions where PAP is elevated as in the FRY1 mutants in *A. thaliana*. Thirdly, we have shown that when DICER1 levels are low, tRF-3s decrease expression of target luciferase reporters in Kuscu et al 2018. Therefore, another possibility is that in cancers where DICER1 is downregulated and miRNA levels are low, any stabilization of tRF-1s would lead to entry into RISC. The functional implications of tRF-1 stabilization will be explored in future studies.

“Another important question is under what conditions is tRF-1 entry into RISC permitted? One such case may be under transient cell states, such as stress. For example, it is known that XRN2 subnuclear localization is altered under heat stress [[75,76]](https://paperpile.com/c/xpEjkr/gVZu+ZtcF). Whether tRF-1s enter RISC and alter expression of heat stress related genes or aid in recovery from heat stress is unknown. Other conditions in which tRF-1s may enter RISC include conditions in which XRN inhibitor PAP is high, similar to what we have shown in Fry1 mutants in plants. There are two human orthologs of Fry1: IMPAD1/BPNT2 and BPNT1. IMPAD1/BPNT2 is localized to the golgi and autosomal recessive mutations in this gene have been shown to cause chondrodysplasia in humans [[77–79]](https://paperpile.com/c/xpEjkr/8BWP+l5fU+ODYF). BPNT1 has not been linked to any genetic disorders to date, however, Bpnt1-/- mice develop severe liver damage which leads to whole-body edema and death [[80]](https://paperpile.com/c/xpEjkr/Gx1N). Whether XRNs are inhibited in IMPAD1 and BPNT1 depleted conditions is unknown, but it is possible that under these conditions tRF-1s are stabilized and contribute to the observed pathologies. Some tRF-1s may also regulate genes associated with suppression of cellular motility because XRN2 has been shown to be required for cellular invasion in glioblastoma cell lines [[81]](https://paperpile.com/c/xpEjkr/qhRh). Finally, tRF-1s may enter Ago2 more efficiently when global levels of miRNAs are diminished. miRNA levels have been shown to be depleted in DICER1 syndrome, a syndrome that predisposes patients to a variety of different malignancies [[82–87]](https://paperpile.com/c/xpEjkr/7XW5+gNLr+3JCk+kiUa+b9HK+HA1i). We have previously shown that tRF-3 mediated repression of a luciferase reporter is enhanced under DICER1 depletion [[6]](https://paperpile.com/c/xpEjkr/f6DS).”

**A few other points for revision or discussion:**

**1. It would be much convenient for the readers if the authors could draw a**

**carton/schematic to show tRNA processing in Figure 1A.**

We agree that this would be helpful for readers. We have added a schematic showing tRNA processing as new Figure 1A.

**Figure 1. tRF-1s are highly unstable.**

A) Schematic of tRNA fragment (tRF) biogenesis. Precursor tRNAs are processed by RNase P, ELAC1/2, and the TSEN complex to give rise to tRF-leaders, tRF-1s, and intron-tRFs, respectively. It is unknown what enzymes produce the majority of tRF-5s and tRF-3s from mature tRNAs.

**2. For Figure 1G, tRF-1001 expression level is relative to miR-21-5p. Why miR-21-5p is**

**chose to perform normalization? How about the relative expressions in Figure 1D/E/F?**

**Are they also relative to miR-21-5p, and why?**

Fig 1D-F is from the small RNA seq data, which is normalized to global small RNA levels.

Figure 1G is a qPCR result that we performed in 293T cells, where we added the normalization control miR-21-5p because it is an abundant, stable miRNA. We have shown miR-21-5p decay kinetics from the same experiment as Figure 1D/E/F (Supp Fig 1C). We have clarified this in the figure and figure legend.

**Supplemental Figure 1.** C) Decay curve for miR-21-5p in HDMYZ cells (sRNA-seq).

**3. In Figure 3A, 1h actD treatment will delete all pre-tRNA serTGA in siCon. But in Figure 3C, after 1h actD treatment, pre-tRNA serTGA can still be detected. How to explain this**

**Contradiction?**

We respectfully disagree with the reviewer. As can be seen from the zero hour time points, Fig 3C is much more exposed than Fig 3A, explaining why we see a weak signal at the 1hr time point in Fig 3C that is not seen in 3A. Therefore, Fig 3A and Fig 3C do not contradict each other. Below we have included a lighter exposure of pre-tRNA SerTGA:

**4. Figure 3D is only one example of XRN2 binding and not really that convincing. Please**

**check XRN2 binding profile around all pre-tRNAs using the eCLIP data.**

We have included this analysis in Supp Fig 3 B-E. We observed the highest number of reads for XRN2 binding globally near the beginning of tRNA trailers for both K562 cells and HepG2 cells. There was no clear pattern of binding of XRN2 to miRNAs. Further evidence for XRN2 binding to tRNA trailers is exemplified in Cortazar et al 2022, which was published while our manuscript has been under review. Cortazar et al 2022 show a strong peak for catalytic dead XRN2 at tRNA trailers.

**Supplemental Figure 3.** B,C) ENCODE XRN2 eCLIP metagene plot for tRNA and miRNA genes, respectively in K562 cells. D,E) Same as in B & C, except in HepG2 cells.

**5. In Figure 3E, expressing catalytic dead XRN2 accumulate more tRF-1s than shXRN2,**

**how to explain this?**

D235A XRN2 is a dominant negative protein, as explained in Fong et al 2015. shXRN2 does not completely remove all XRN2 protein, even though the western blot makes it look like it is because of the poor sensitivity of WBs. Thus it is not surprising that overexpressing D235A XRN2 will further decrease the activity of any endogenous XRN2 that persists after shXRN2. This additive effect is responsible for the higher level of tRF-1001 in that lane. We have clarified this in the text:

“Expression of shXRN2 and D235A XRN2 resulted in an even higher accumulation of tRF-1001 due to the additive effects of this condition. D235A XRN2 is a dominant negative protein [[28]](https://paperpile.com/c/xpEjkr/AUG6), therefore overexpressing D235A XRN2 will further decrease the activity of any endogenous XRN2 that persists after shXRN2.”

**6. “1 was a miRNA, 1 mapped to a piRNA locus, 1 was a misc-tRF, 3 were intron-tRFs,**

**and 25 were tRF-1s.” It would be better to use word rather than number, if the single**

**digit is subject.**

We have changed this in the text:

“Out of the 31 small RNAs that significantly increased in both datasets, one was a miRNA, one mapped to a piRNA locus, one was a misc-tRF, three were intron-tRFs, and twenty-five were tRF-1s.”

**7. Since you introduce Figure 4E before Figure 4D, it would be better to change the order**

**of 4E and 4D.**

We have changed this in the figure numbering and text.

**8. For Figure 5 legend, it should be “E, G) Scatter plot” rather than “E, F) Scatter plot”.**

We have changed this in the text.

**9. Figure 6 model was not discussed in the body paragraphs.**

We now referenced Figure 6 in the body paragraphs:

“In this study, we found that tRF-1s are likely excluded from RISC due to a very short half-life mediated by XRN2 activity (Figure 6)”
